# Differential genotoxicity of diphenyl diselenide (PhSe)_2_ and diphenyl ditelluride (PhTe)_2_

**DOI:** 10.7717/peerj.290

**Published:** 2014-03-18

**Authors:** Daiane Francine Meinerz, Josiane Allebrandt, Douglas O.C. Mariano, Emily P. Waczuk, Felix Antunes Soares, Waseem Hassan, João Batista T. Rocha

**Affiliations:** Departamento de Bioquímica e Biologia Molecular, Centro de Ciências Naturais e Exatas, Universidade Federal de Santa Maria, Santa Maria, RS, Brasil

**Keywords:** Organoselenium, Genotoxicity and mutagenicity, Organotellurium

## Abstract

Organoselenium compounds have been pointed out as therapeutic agents. In contrast, the potential therapeutic aspects of tellurides have not yet been demonstrated. The present study evaluated the comparative toxicological effects of diphenyl diselenide (PhSe)_2_ and diphenyl ditelluride (PhTe)_2_ in mice after *in vivo* administration. Genotoxicity (as determined by comet assay) and mutagenicicity were used as end-points of toxicity. Subcutaneous administration of high doses of (PhSe)_2_ or (PhTe)_2_ (500 µmol/kg) caused distinct genotoxicity in mice. (PhSe)_2_ significantly decreased the DNA damage index after 48 and 96 h of its injection (*p* < 0.05). In contrast, (PhTe) caused a significant increase in DNA damage (*p* < 0.05) after 48 and 96 h of intoxication. (PhSe)_2_ did not cause mutagenicity but (PhTe)_2_ increased the micronuclei frequency, indicating its mutagenic potential. The present study demonstrated that acute *in vivo* exposure to ditelluride caused genotoxicity in mice, which may be associated with pro-oxidant effects of diphenyl ditelluride. In addition, the use of this compound and possibly other related tellurides must be carefully controlled.

## Introduction

**Figure 1:**
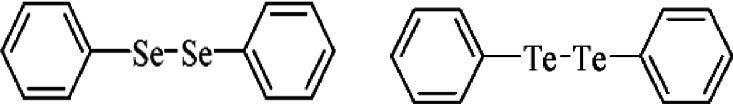
Structure of diphenyl diselenide and diphenyl ditelluride.

Selenium (Se) and Tellurium (Te) belongs to the chalcogen family, sharing similar electronic configuration and some chemical properties with sulfur (S) (Comasseto et al., 1997; Comasseto, 2010). Se has a fundamental role in several living organisms as component of several antioxidant enzymes, including glutathione peroxidase and thioredoxin reductase (Arner & Holmgren, 2000; Nogueira & Rocha, 2011). Despite its biological role, the excess of selenium can be toxic due its ability to generate free radicals and catalyze thiol oxidation (Barbosa et al., 1998; Nogueira, Zen & Rocha, 2004; Rocha et al., 2012; Hassan & Rocha, 2012; Kade, Balogun & Rocha, 2013). The excess of free radical formation can damage mammalian tissues including thiol containing enzymes that are sensitive to pro-oxidant situations (Rocha et al., 2012; Rosa et al., 2007; Maciel et al., 2000). Diphenyl diselenide (PhSe)_2_, (Fig. 1) is a simple and stable organoselenium compound widely used in organic synthesis and it has been proposed as a good candidate for pharmacological and therapeutic purposes (Nogueira, Zen & Rocha, 2004; Rosa et al., 2007; Nogueira & Rocha, 2011). (PhSe)_2_ exhibits thiol peroxidase-like activity superior to that of ebselen, an organoselenium compound that has been used in clinical trials as antioxidant and mimetic of native glutathione peroxidase enzymes (Nogueira & Rocha, 2011; Kade & da Rocha, 2013; Kade, Balogun & Rocha, 2013). However, exposure to high doses of (PhSe)_2_ can deplete thiols in different tissues and can be neurotoxic to rodents (Maciel et al., 2000). The LD50 of diphenyl diselenide is 210 µmol/kg (intraperitoneal) or greater than 500 µmol/kg (subcutaneous) in adult mice (Nogueira et al., 2003).

There are reports that trace amounts of Te are present in body fluids such as blood and urine (Chasteen et al., 2009). Te has also been found in the form of tellurocysteine and telluromethionine in several proteins in bacteria, yeast and fungi but telluroproteins have not been identified in animal cells (Bienert, Schussler & Jahn, 2008). Thus, in contrast to selenium, tellurium does not have physiological functions (Taylor, 1996). Literature has demonstrated immunomodulatory, antioxidant and anticancer properties of various organotellurides (Nogueira, Zen & Rocha, 2004; Avila et al., 2012), semisynthetic tellurosubtilisin (Mao et al., 2005) and dendrimeric organotellurides (Francavilla et al., 2001). More sophisticated telluride molecules were synthesized from polystyrene nanoparticle via microemulsion polymerization. The nanoenzyme showed higher efficiency and provided a platform for the synthesis and designing of polymeric nanoparticles as excellent model of enzyme mimics (Huang et al., 2008). Organotellurium compounds can also mimic glutathione peroxidase activity (Engman et al., 1995) and, consequently, these compounds can be potential antioxidants, effective against hydrogen peroxide, peroxynitrite, hydroxyl radicals and superoxide anions (Andersson et al., 1994; Kanski et al., 2001; Jacob et al., 2000).

Recently, our research group demonstrated that organoselenium and organotellurium present hemolytic and genotoxic effects in human blood cells (Santos et al., 2009a; Santos et al., 2009b; Caeran Bueno et al., 2013), which is in accordance with results published by other laboratories in experimental bacteria and rodent models (Degrandi et al., 2010). Similarly, organoselenides and tellurides can be toxic in different *in vivo* and *in vitro* models of animal pathologies (Maciel et al., 2000; Taylor, 1996; Stangherlin, Rocha & Nogueira, 2009; Moretto et al., 2007; Heimfarth et al., 2011; Heimfarth et al., 2012a; Heimfarth et al., 2012b; Comparsi et al., 2012). In effect, diphenyl ditelluride (PhTe)_2_ was found to be extremely toxic to mice and rats after acute or chronic exposure (Maciel et al., 2000; Heimfarth et al., 2012b; Comparsi et al., 2012). The toxicity of tellurides can be associated with their pro-oxidant activity, particularly, the oxidation of thiol- and selenol-groups of proteins (Nogueira, Zen & Rocha, 2004; Comparsi et al., 2012; Hassan & Rocha, 2012).

Following our interest to determine the boundary between the potential protective and toxic properties of organochalcogens, the present study was designed to evaluate the toxic potential of (PhSe)_2_ and (PhTe)_2_ in mice. We have determined the genotoxicity and mutagenicity of these compounds after acute administration to Swiss male mice, using DNA damage and micronuclei frequency as end-points of toxicity.

## Material and Methods

### Chemicals

The chemical structure of organochalcogens tested in this study is shown in Fig. 1 diphenyl diselenide and diphenyl ditelluride. The compounds were dissolved in canola oil immediately before use. (PhSe)_2_ and (PhTe)_2_ were obtained from Sigma-Aldrich. All other chemicals were of analytical grade and obtained from standard commercial suppliers.

### Animals

Male Swiss adult mice weighing 30–40 g were obtained from our own breeding colony (Animal house-holding, UFSM-Brazil). Animals were kept in separate animal cages, on a 12-h light/dark cycle, at a room temperature of (23 °C ± 3) and with free access to food and water. The animals were used according to the guidelines of the committee on care and use of experimental animal resources of the Federal University Of Santa Maria, Brazil (23081.002435/2007-16).

Mice were divided in six groups (*n* = 5) and received one subcutaneous injection of (1) canola oil (Control group 48 h, mice were euthanized 48 h after the oil injection); (2) diphenyl ditelluride (500 µmol/kg in canola oil, euthanized 48 h after injection) ; (3) diphenyl diselenide (500 µmol/kg in canola oil, euthanized 48 h after injection); (4) canola oil (Control group 96 h, mice were euthanized 96 h after injection); (5) diphenyl ditelluride (500 µmol/kg in canola oil, euthanized 96 h after injection) and (6) diphenyl diselenide (500 µmol/kg in canola oil, euthanized 96 h after injection). The doses were based in a previous acute toxicological study by Maciel et al. (2000).

### Sample preparation for comet assay

Mice were anesthesized with ketamine and 2.5 ml blood samples were collected by heart puncture and immediately euthanized by decaptation. Mice blood leukocytes were isolated and used in the comet test but no pre-incubation was carried out (Santos et al., 2009a; Santos et al., 2009b; Meinerz et al., 2011).

### Micronucleus test

In a micronucleus test (MN), two samples of blood from each animal were placed in a microscope slides and air dried at room temperature. Slides were stained with 5% May-Grunwald-Giemsa for 5 min. The criteria used for the identification of MN were a size smaller than one-third of the main nucleus, no attachment to the main nucleus, and identical color and intensity as in the main nucleus. MN were counted in 2000 cells with well-preserved cytoplasm and calculated as: % MN = number of cells containing micronucleus × 100/total number of cells counted. Micronuclei presence was determined by three investigators that were blind to the animal treatments.

### Comet assay

**Figure 2:**
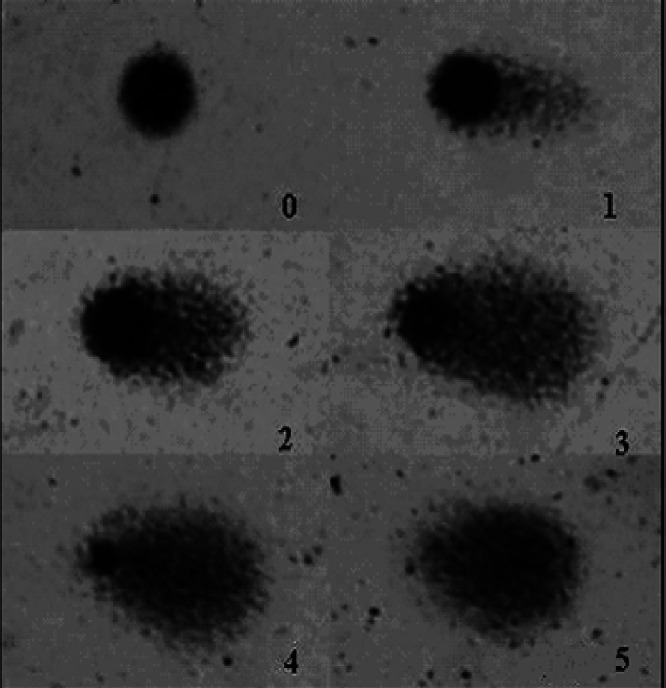
DNA damage quantification. Classifications of DNA damage in human leukocytes. DNA damage index was calculated from cells in different damage levels, which were classified in the visual score by the measurement of DNA migration length and in the amount of DNA in the tail. The level 5 was excluded from our evaluation.

Comet assay is a rapid, simple and sensitive technique for measuring DNA breaks in single cells. This test has been used to investigate the effect of many toxic agents on DNA (Collins & Harrington, 2002; Blasiak, Arabski & Krupa, 2004). The comet assay was performed under alkaline conditions according to the procedures described by Santos et al. (2009a) and Santos et al. (2009b). The slides obtained from white blood cells of treated mice were analyzed under blind conditions by at least two individuals. DNA damage is presented as DNA damage index (DI). The DNA damage was calculated from cells in different damage classes (completely undamaged: 100 cells × 0 to maximum damaged −100 cells × 4). Damage index is illustrated in Fig. 2 and classes were determined considering the DNA tail and DNA migration length.

### Statistical analysis

Data are expressed as mean ± SD from five independent experiments performed in duplicate or triplicate. Statistical analysis was performed using a Kruskal-Wallis Test followed by Dun’s test. Results were considered statistically significant when *p* < 0.05.

## Results

No animal died during the experimental period. After 48 h of diselenide or ditelluride treatment, mice did not show symptoms of toxicity such as stereotypical behavior, ataxia, diarrhea, increased dieresis or abdominal writings. However, after 96 h, the group treated with (PhTe)_2_ presented diarrhea, low level of motor activity and a decrease in body weight (data not shown); which is in accordance with previous finding from our laboratory (Maciel et al., 2000).

### Comet assay

After *in vivo* administration, diphenyl diselenide caused a significant decrease in DNA damage index (DI) both after 48 and 96 h. In contrast, diphenyl ditelluride caused a significant increase in DNA damage index (DI). After 48 h, the damage caused by ditelluride was about 25 and 100% higher than control and diphenyl diselenide groups, respectively (Table 1). After 96 h, the DI caused by diphenyl ditelluride was about 30 and 90% higher than control and diselenide treated mice, respectively (Table 1).

**Table 1:** DNA damage levels in leukocytes from mice treated with diselenide or ditelluride.

Compound	Hours of exposition	Damage levels of DNA					DI
		0	1	2	3	4	
**Control**	**48 h**	61.0 ± 0.5	19.6 ± 2.0	13.4 ± 1.4	4.5 ± 0.8	1.0 ± 0.5	63.0 ± 2.5^a^
**(PhSe)_2_**	**48 h**	77.2 ± 3.6	11.8 ± 1.6	6.6 ± 1.3	3.8 ± 1.1	0.6 ± 0.2	40.8 ± 7.8^b^
**(PhTe)_2_**	**48 h**	48.0 ± 9.7	32.3 ± 9.6	13.0 ± 3.2	5.0 ± 1.0	1.6 ± 0.6	80.0 ± 9.3^c^
**Control**	**96 h**	63.5 ± 0.5	20.7 ± 6.5	12.5 ± 5.5	3.7 ± 0.5	0.0 ± 0.0	58.0 ± 4.6^a^
**(PhSe)_2_**	**96 h**	80.0 ± 2.0	10.0 ± 2.0	5.0 ± 3.0	3.0 ± 0.6	2.0 ± 2.0	40.0 ± 1.1^b^
**(PhTe)_2_**	**96 h**	59.5 ± 3.5	19.0 ± 7.0	12.0 ± 3.0	9.2 ± 0.8	1.6 ± 0.5	76.0 ± 1.2^c^

**Notes.**

Distribution of damage levels in mice leukocytes exposed to diphenyl diselenide and diphenyl ditelluride (500 µmol/kg, s.c.). DNA damage is presented as DNA damage index (DI). Data are expressed as means for five independent experiments. Statistical analysis by a Kruskal-Wallis Test test followed by Dun’s test.

### Micronucleus test

**Figure 3:**
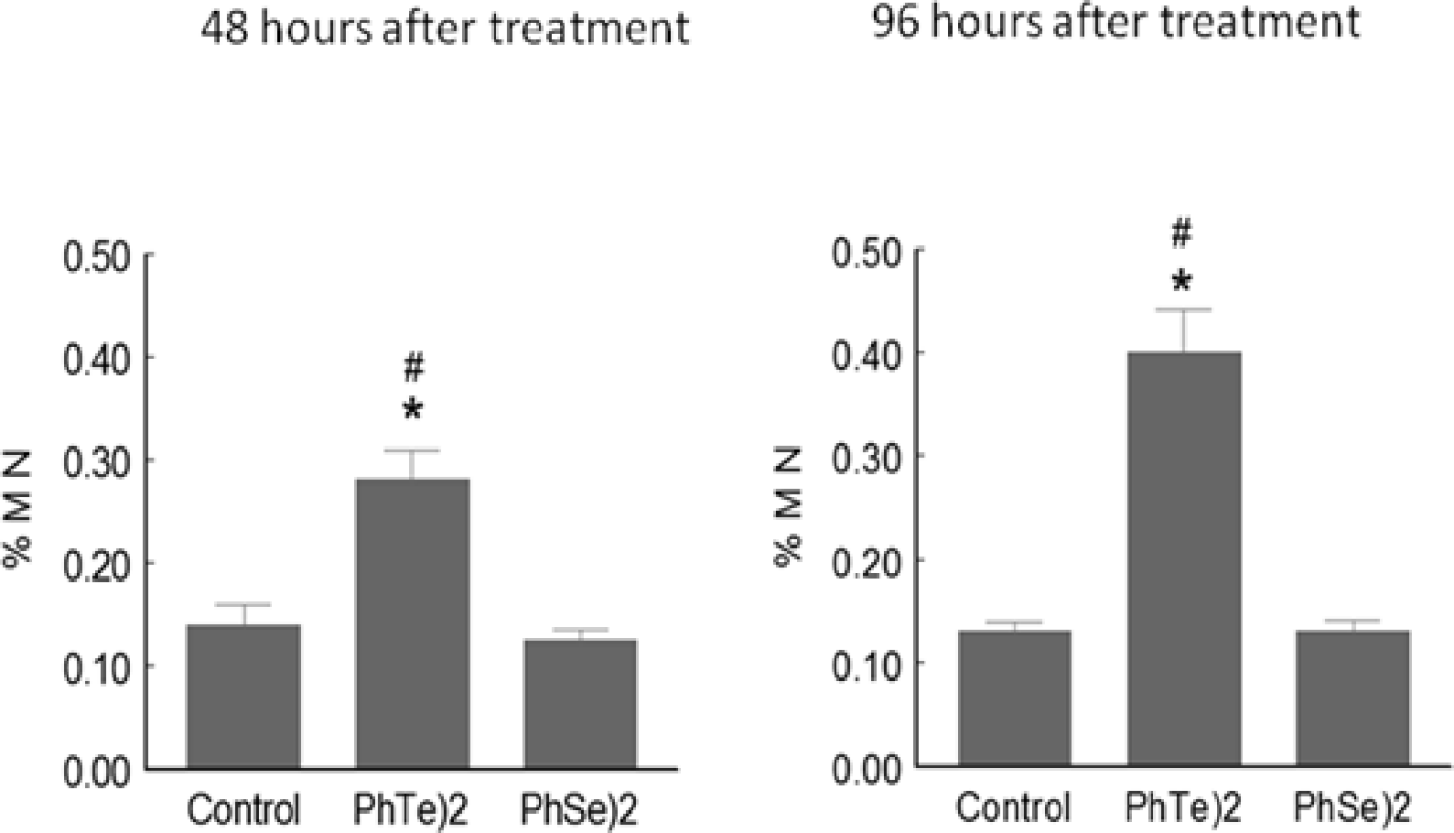
Micronuclei frequency after treatment with diselenide and ditelluride. Frequency of Micronuclei (MN) cells in mice exposed to (PhTe)_2_ or (PhSe)_2_. Mice were exposed to a single dose of diselenide or ditelluride (500 µmol/kg, s.c.). Forty eight and 96 h after the injection, blood cells were examined for the presence of micronuclei. Data are expressed as mean ± SD for 5 mice per group. ∗ denoted *p* > 0.01 as compared to control group; # Denoted *p* > 0.01 as compared to diphenyl diselenide.

After 48 or 96 h of a single dose of diphenyl ditelluride, there was a significant increase in the number of micronuclei in mice when compared with control and diphenyl diselenide group (Fig. 3). Diphenyl diselenide did not modify the number of micronuclei when compared to the control group (Fig. 3).

## Discussion

The selected dose of both chalcogens was based on our previous report (Maciel et al., 2000), where we tested different doses for acute and chronic exposure. Similarly, in the same dose range, diphenyl diselenide has been reported to have interesting pharmacological effects, such as antinoception and anti-inflammatory effects, among others, (see, for instance, Savegnago et al., 2008; Savegnago et al., 2007a; Savegnago et al., 2007b and Savegnago et al., 2006). However, it must be emphasized here that in this range of doses, it also causes toxicity in mice and rats (Nogueira et al., 2003; Nogueira & Rocha, 2011). Consequently, the acute use of diphenyl diselenide may be possible, but its chronic or repeated use is unfeasible.

The results presented here indicate clear toxic effects of (PhTe)_2_ when compared with (PhSe)_2_. Tellurium (Te) has the potential of redox cycling which leads to formation of reactive oxygen species (ROS) which can damage biomolecules (Maciel et al., 2000; Nogueira, Zen & Rocha, 2004; Santos et al., 2009a; Santos et al., 2009b; Degrandi et al., 2010; Sailer et al., 2004; Caeran Bueno et al., 2013). Organotellurium-induced intracellular ROS accumulation has been reported to be the cause of cell death in HL-60 and different types of cancer cells (McNaughton et al., 2004; Sandoval et al., 2010; Ding et al., 2002; Rigobello et al., 2009). In contrast, exposure of mice to (PhSe)_2_ caused a significant decrease in the DNA damage index (DI) both after 48 and 96 h of drug administration as shown in Table 1. The protective effect can be attributed to its antioxidant or GPx like activity (Nogueira & Rocha, 2011).

As observed in DNA damage test, the toxic behavior of (PhTe)_2_ was completely different than (PhSe)_2_ in micronucleus assay. The frequency of mutations, showed by an increase of micronuclei frequency, reinforce the toxicity of (PhTe)_2_. It is important to note that (PhSe)_2_ did not modify the number of micronuclei, when compared to the control group (Fig. 3). Previous studies have also demonstrated mutagenicicity of (PhTe)_2_ at higher concentrations in V79 cells (Rosa et al., 2007). We have also reported the mutagenicity of another Te-containing organic compound, (*S*)-dimethyl 2-(3-(phenyltellanyl) propanamido) succinate in mice leukocytes (Meinerz et al., 2011)

In conclusion, the results presented here indicate that diphenyl ditelluride is toxic to mice, whereas at the same dose diphenyl diselenide had protective effects. These effects may be linked to the pro-oxidant activity exhibited by organotellurium compounds. This data supports studies that have been published about the toxicological and pharmacological effects of organochalcogens in different pathological models. In effect, our data indicated that diphenyl diselenide can have protective effects after *in vivo* administration to mice, which can be related to its antioxidant properties, whereas diphenyl ditelluride is much more toxic than diphenyl diselenide. Furthermore, in view of the genotoxic effect of (PhTe)_2_, the indication in the literature that organotellurides could be therapeutically active compounds must be revisited taking into consideration the potential toxicity of this element. Accordingly, additional studies will be needed to elucidate the mechanism(s) by which (PhTe)_2_ mediates its toxicity and whether or not distinct chemical forms of organotellurides can have a similar toxic effect in animal models.

## References

[ref-1] Andersson CM, Brattsand R, Hallberg A, Engman L, Persson J, Moldéus P, Cotgreave I (1994). Diaryl tellurides as inhibitors of lipid peroxidation in biological and chemical systems. Free Radical Research.

[ref-2] Arner ES, Holmgren P (2000). Physiological functions of thioredoxin and thioredoxin reductase. European Journal of Biochemistry.

[ref-3] Avila DS, Benedetto A, Au C, Manarin F, Erikson K, Soares FA, Rocha JBT, Aschner M (2012). Organotellurium and organoselenium compounds attenuate Mn-induced toxicity in Caenorhabditis elegans by preventing oxidative stress. Free Radical Biology and Medicine.

[ref-4] Barbosa NB, Rocha JBT, Zeni G, Emanuelli T, Beque MC, Braga AL (1998). Effect of organic forms of selenium on *δ*-aminolevulinate dehydratase from liver, kidney, and brain of adult rats. Toxicology and Applied Pharmacology.

[ref-5] Bienert GP, Schussler MD, Jahn TP (2008). Metalloids: essential, beneficial or toxic? Major intrinsic proteins sort it out. Trends in Biochemical Sciences.

[ref-6] Blasiak J, Arabski M, Krupa R, Wozniak K, Rykala J, Kolacinska A, Morawiec Z, Drzewoski J, Zadrozny M (2004). Basal, oxidative and alkalative DNA damage, DNA repair efficacy and mutagen sensitivity in breast cancer. Mutation Research.

[ref-7] Caeran Bueno D, Meinerz DF, Allebrandt J, Waczuk EP, dos Santos DB, Mariano DOC, Rocha JBT (2013). Cytotoxicity and genotoxicity evaluation of organochalcogens in human leucocytes: a comparative study between ebselen, diphenyl diselenide, and diphenyl ditelluride. BioMed Research International.

[ref-8] Chasteen TG, Fuentes DE, Tantalean TC, Vasquez CC (2009). Tellurite: history, oxidative stress, and molecular mechanisms of resistance. FEMS Microbiology Reviews.

[ref-9] Collins AR, Harrington V (2002). Repair of oxidative DNA damage: assessing its contribution to cancer prevention. Mutagenisis.

[ref-10] Comasseto JV (2010). Selenium and tellurium chemistry: historical background. Journal of the Brazilian Chemical Society.

[ref-11] Comasseto JV, Ling LW, Petragnani N, Stefani HA (1997). Vinylic selenides and tellurides/preparation, reactivity and synthetic compounds. Synthesis.

[ref-12] Comparsi B, Meinerz DF, Franco JL, Posser T, de Souza Prestes A, Stefanello ST, dos Santos DB, Wagner C, Farina M, Aschner M, Dafre AL, Rocha JB (2012). Diphenyl ditelluride targets brain selenoproteins *in vivo*: inhibition of cerebral thioredoxin reductase and glutathione peroxidase in mice after acute exposure. Molecular and Cellular Biochemistry.

[ref-13] Degrandi TH, de Oliveira IM, D’Almeida GM, Garcia CRL, Villela IV, Guecheva TN, Rosa RM, Henriques JAP (2010). Evaluation of the cytotoxicity, genotoxicity and mutagenicity of diphenyl ditelluride in several biological models. Mutagenesis.

[ref-14] Ding DW, Hasegawa T, Peng D, Hosaka H, Seko Y (2002). Preliminary investigation on the cytotoxicity of tellurite to cultured HeLa cells. Journal of Trace Elements in Medicine and Biology.

[ref-15] Engman L, Person J, Vessman K, Ekstrom M, Berglund M, Andersson CM (1995). Organotellurium compounds as efficient retarders of lipid peroxidation in methanol. Free Radical Biology and Medicine.

[ref-16] Francavilla C, Drake MD, Bright FV, Detty MR (2001). Dendrimeric organochalcogen catalysts for the activation of hydrogen peroxide: improved catalytic activity through statistical effects and cooperativity in successive generations. Journal of the American Chemical Society.

[ref-17] Hassan W, Rocha JBT (2012). Interaction profile of diphenyl diselenide with pharmacologically significant thiols. Molecules.

[ref-18] Heimfarth L, Loureiro SO, Reis KP, de Lima BO, Zamboni F, Lacerda S, Soska AK, Wild L, da Rocha JBT, Pessoa-Pureur R (2012a). Diphenyl ditelluride induces hypophosphorylation of intermediate filaments through modulation of DARPP-32-dependent pathways in cerebral cortex of young rats. Archives of Toxicology.

[ref-19] Heimfarth L, Loureiro SO, Dutra MF, Andrade C, Pettenuzzo L, Guma FT, Gonçalves CA, da Rocha JB, Pessoa-Pureur R (2012b). In vivo treatment with diphenyl ditelluride induces neurodegeneration in striatum of young rats: implications of MAPK and Akt pathways. Toxicology and Applied Pharmacology.

[ref-20] Heimfarth L, Loureiro SO, Reis KP, de Lima BO, Zamboni F, Gandolfi T, Narvaes R, da Rocha JBT, Pessoa-Pureur R (2011). Cross-talk among intracellular signaling pathways mediates the diphenyl ditelluride actions on the hippocampal cytoskeleton of young rats. Chemical Research in Toxicology.

[ref-21] Huang X, Liu Y, Liang K, Tang Y, Liu J (2008). Construction of the active site of gluthathione peroxidase on polymer-based nanoparticles. Biomacromolecules.

[ref-22] Jacob C, Arteel GE, Kanda T, Engman L, Sies H (2000). Water soluble organotellurium compounds: catalytic protection against peroxynitrite and release of zinc from metallothionein. Chemical Research in Toxicology.

[ref-23] Kade IJ, Balogun BD, Rocha JBT (2013). In vitro glutathione peroxidase mimcry of ebselen is linked to its oxidation of critical thiols on key cerebral suphydryl proteins-a novel component of its gpx-mimic antioxidant mechanism emerging from its thiol-modulated toxicology and pharmacology. Chemico-Biological Interactions.

[ref-24] Kade IJ, da Rocha JBT (2013). Pharmacology of organoselenium compounds: emphasis on puzzling mechanistic switching from their glutathione peroxidase mimic in vivo. Biokemistri.

[ref-25] Kanski J, Drake J, Aksenova M, Engman L, Butterfield DA (2001). Antioxidant activity of the organotellurium compound 3-[4-(N,N-dimethylamino) benzenetellurenyl] propanesulfonic acid against oxidative stress in synaptosomal membrane systems and neuronal cultures. Brain Research.

[ref-26] Maciel EN, Bolzan RC, Braga AL, Rocha JBT (2000). Diphenyl diselenide and diphenyl ditelluride differentially affect *δ*-aminolevulinate dehydratase from liver, kidney, and brain of mice. Journal of Biochemical and Molecular Toxicology.

[ref-27] Mao SZ, Dong ZY, Liu JQ, Li XQ, Liu XM, Luo GM, Shen JC (2005). Semisynthethic tellurosubtilisin with gluthathione peroxidase activity. Journal of American Chemical Society.

[ref-28] McNaughton M, Engman L, Birmingham A, Powis G, Cotgreave IA (2004). Cyclodextrin-derived diorganyl tellurides as glutathione peroxidase mimics and inhibitors of thioredoxin reductase and cancer cell growth. Journal of Medicinal Chemistry.

[ref-29] Meinerz DF, Sudati JH, Santos DB, Frediani A, Alberto EE, Allebrandt J, Franco JL, Barbosa NBV, Aschner M, Rocha JBT (2011). Evaluation of the biological effects of (*S*)-dimethyl 2-(3-(phenyltellanyl) propanamido) succinate, a new telluroamino acid derivative of aspartic acid. Achieves of Toxicology.

[ref-30] Moretto MB, Boff B, Franco J, Posser T, Roessler TM, Souza DO, Nogueira CW, Wofchuk S, Rocha JBT (2007). Ca(2p) influx in rat brain: effect of diorganylchalcogenides compounds. Toxicological Sciences.

[ref-31] Nogueira CW, Meotti FC, Curte E, Pilissão C, Zeni G, Rocha JBT (2003). Investigations into the potential neurotoxicity induced by diselenides in mice and rats. Toxicology.

[ref-32] Nogueira CW, Rocha JBT (2011). Toxicology and pharmacology of selenium: emphasis onsynthetic organoselenium compounds. Archives of Toxicology.

[ref-33] Nogueira CW, Zen G, Rocha JBT (2004). Organoselenium and organotellurium compounds: toxicology and pharmacology. Chemical Reviews.

[ref-34] Rigobello MP, Gandin V, Folda A, Rundlo AK, Fernandes FAP, Bindoli A, Marzano C, Bjornstedt M (2009). Treatment of human cancer cells with selenite or tellurite in combination with auranofin enhances cell death due to redox shift. Free Radical Biology and Medicine.

[ref-35] Rocha JBT, Saraiva RA, Garcia SA, Gravina F, Nogueira CW (2012). Aminolevulinate dehydratase (*δ*-ALA-D) as marker protein of intoxication with metals and other pro-oxidant situations. Toxicology Research.

[ref-36] Rosa RM, Hoch NC, Furtado GV, Saffi J, Henriques JAP (2007). DNA damage in tissues and organs of mice treated with diphenyl diselenide. Mutation Research.

[ref-37] Sailer BL, Liles N, Dickerson S, Sumners S, Chasteen TG (2004). Organotellurium compound toxicity in a promyelocytic cell line compared to non-tellurium-containing organic analogue. Toxicology In Vitro.

[ref-38] Sandoval JM, Levêque P, Gallez B, Vásquez CC, Buc Calderon P (2010). Tellurite-induced oxidative stress leads to cell death of murine hepatocarcinoma cells. Biometals.

[ref-39] Santos DB, Schiar VPP, Paixão MW, Meinerz DF, Nogueira CW, Aschner M, Rocha JBT, Barbosa NBV (2009a). Hemolytic and genotoxic evaluation of organochalcogens in human blood cells in vitro. Toxicology in Vitro.

[ref-40] Santos DB, Schiar VPP, Ribeiro MCP, Schwab RS, Meinerz DF, Allebrandt J, Rocha JBT, Nogueira CW, Aschner M, Barbosa NBV (2009b). Genotoxicity of organoselenium compounds in human leukocytes *in vitro*. Mutation Research.

[ref-41] Savegnago L, Jesse CR, Pinto LG, Rocha JB, Nogueira CW (2007a). Diphenyl diselenide attenuates acute thermal hyperalgesia and persistent inflammatory and neuropathic pain behavior in mice. Brain Research.

[ref-42] Savegnago L, Pinto LG, Jesse CR, Rocha JB, Nogueira CW, Zeni G (2007b). Spinal mechanisms of antinociceptive action caused by diphenyl diselenide. Brain Research.

[ref-43] Savegnago L, Jesse CR, Santos AR, Rocha JB, Nogueira CW (2008). Mechanisms involved in the antinociceptive effect caused by diphenyl diselenide in the formalin test. Journal of Pharmacy and Pharmacology.

[ref-44] Savegnago L, Trevisan M, Alves D, Rocha JB, Nogueira CW, Zeni G (2006). Antisecretory and antiulcer effects of diphenyl diselenide. Environmental Toxicology and Pharmacology.

[ref-45] Stangherlin EC, Rocha JBT, Nogueira CW (2009). Diphenyl ditelluride impairs short term memory and alters neurochemical parameters in young rats. Pharmacology, Biochemistry and Behavior.

[ref-46] Taylor A (1996). Biochemistry of tellurium. Biological Trace Element Research.

